# Supplementary figures and images for: ToRQuEMaDA: tool for retrieving queried Eubacteria, metadata and dereplicating assemblies
Source: PeerJ. 2021 May 5;9:e11348. doi: 10.7717/peerj.11348 (PMC8106394; doi:10.7717/peerj.11348)

Tree scale: 0.1

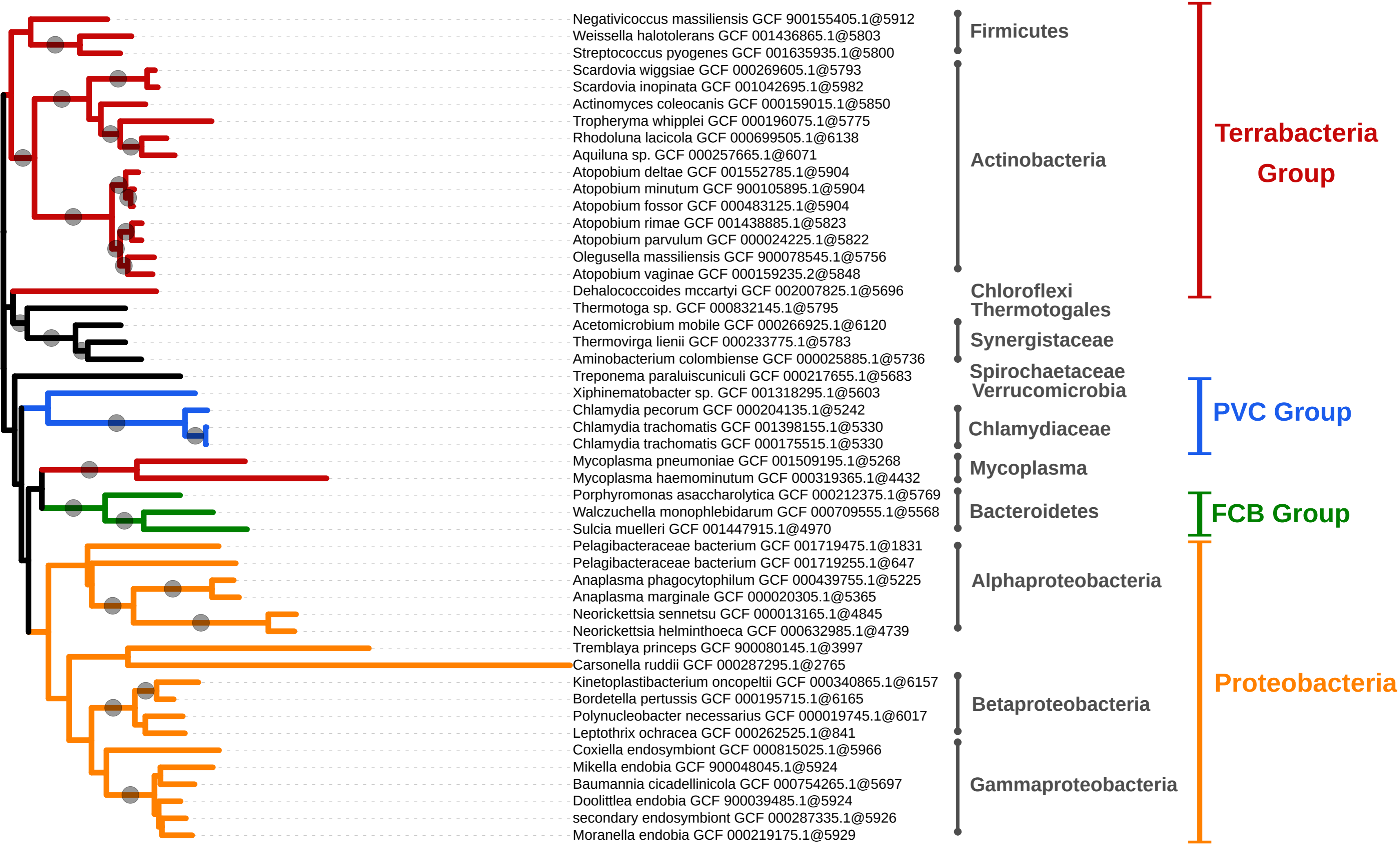

Supplement: Figure S1 — Tree inferred from a supermatrix of concatenated ribosomal proteins (Table 2, A) under the LG4X model using IQ-TREE. Dots on branches indicate maximum bootstrap support values (100%). [file peerj-09-11348-s001.pdf]

Tree scale: 0.1

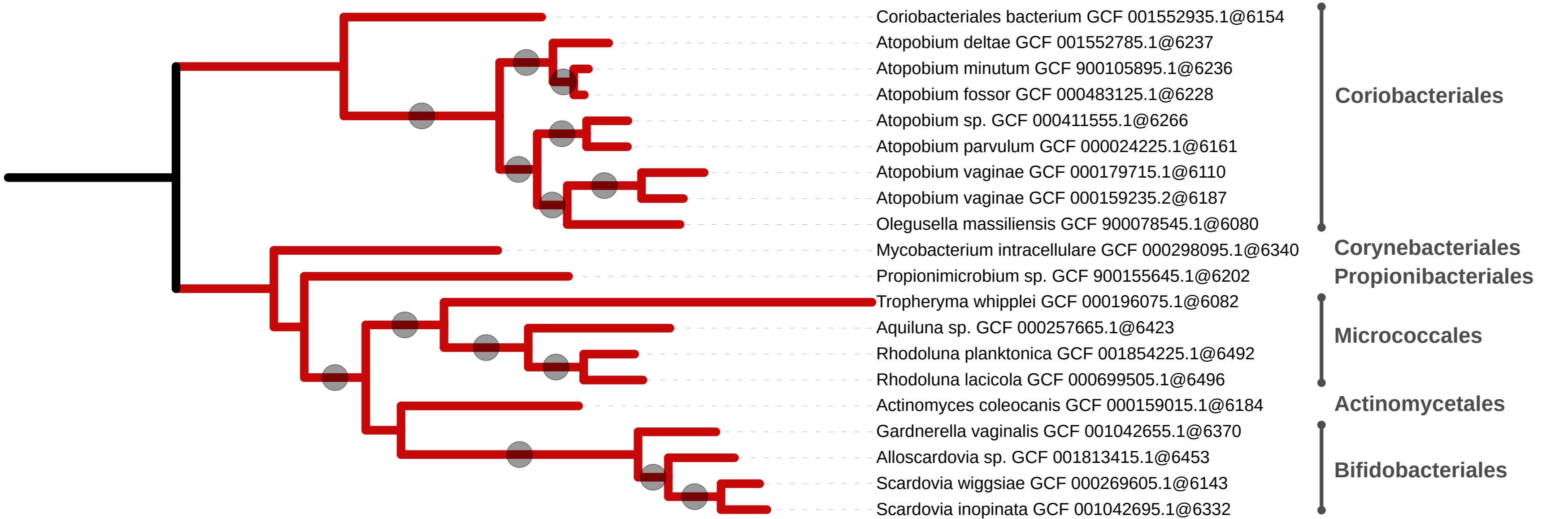

Supplement: Figure S2 — Tree inferred from a supermatrix of concatenated ribosomal proteins (Table 2, C) under the LG4X model using IQ-TREE. Dots on branches indicate maximum bootstrap support values (100%). [file peerj-09-11348-s002.pdf]

Tree scale: 0.1

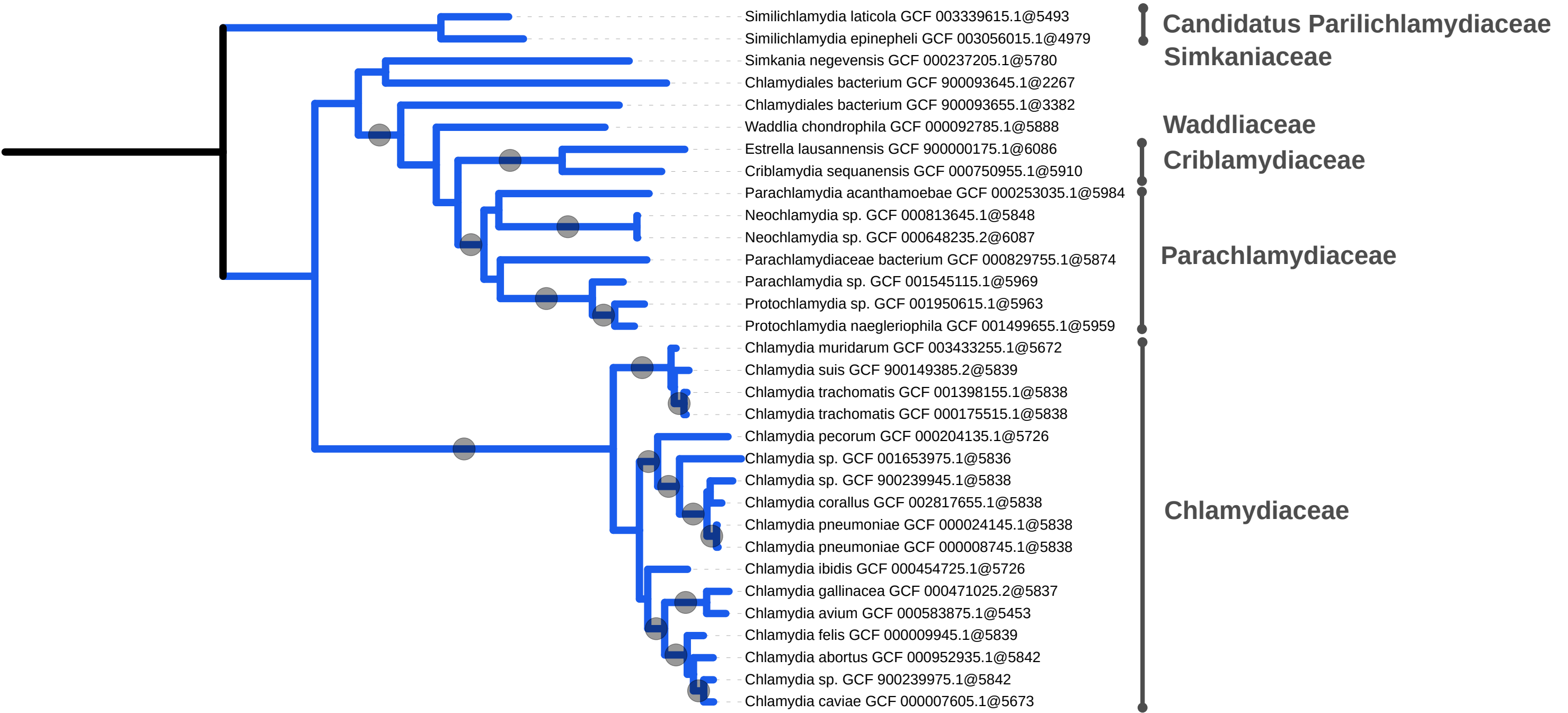

Supplement: Figure S4 — Tree inferred from a supermatrix of concatenated ribosomal proteins (Table 2, E) under the LG4X model using IQ-TREE. Dots on branches indicate maximum bootstrap support values (100%). [file peerj-09-11348-s004.pdf]

Tree scale: 0.01

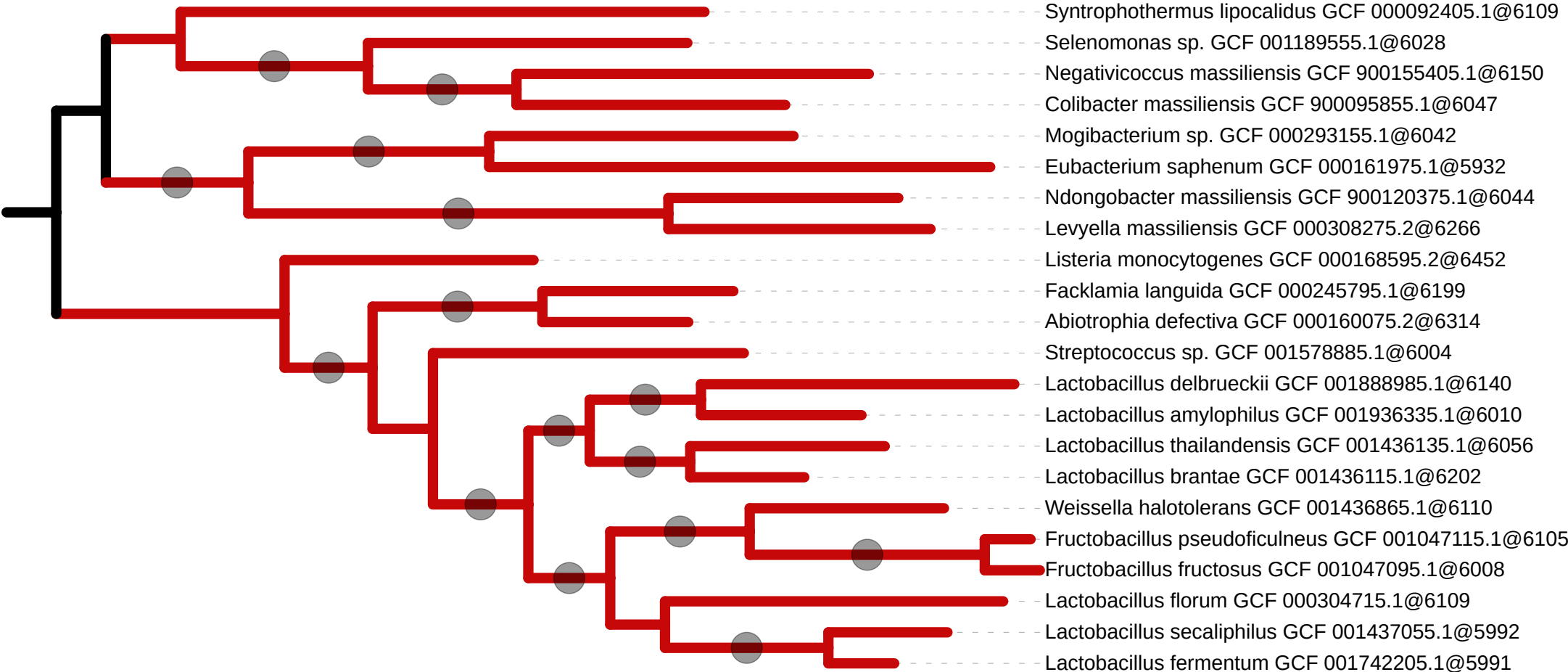

Negativicutes

Clostridia

Bacilli

Supplement: Figure S6 — Tree inferred from a supermatrix of concatenated ribosomal proteins (Table 2, G) under the LG4X model using IQ-TREE. Dots on branches indicate maximum bootstrap support values (100%). [file peerj-09-11348-s006.pdf]

Tree scale: 0.1

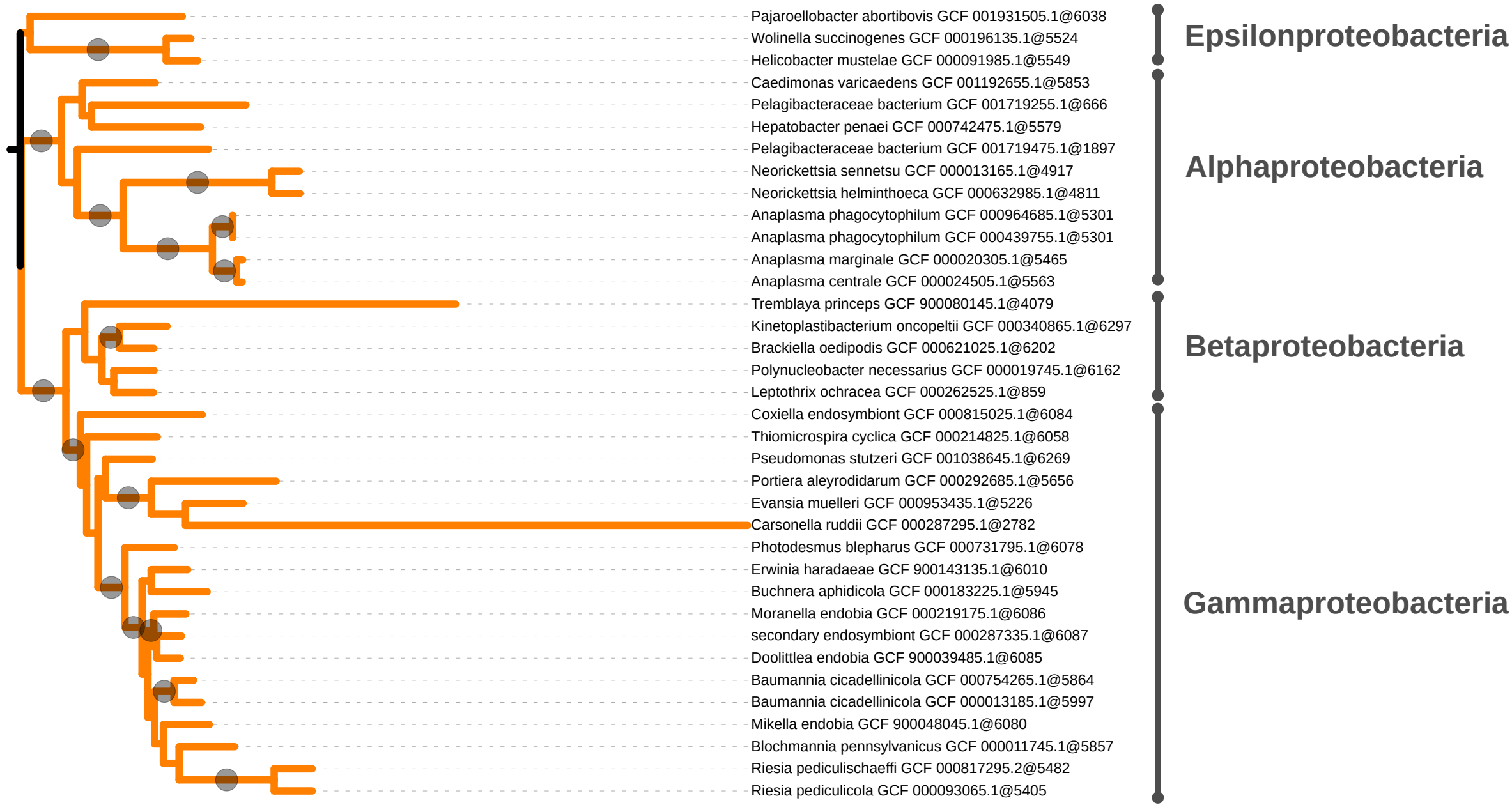

Supplement: Figure S7 — Tree inferred from a supermatrix of concatenated ribosomal proteins (Table 2, H) under the LG4X model using IQ-TREE. Dots on branches indicate maximum bootstrap support values (100%). [file peerj-09-11348-s007.pdf]

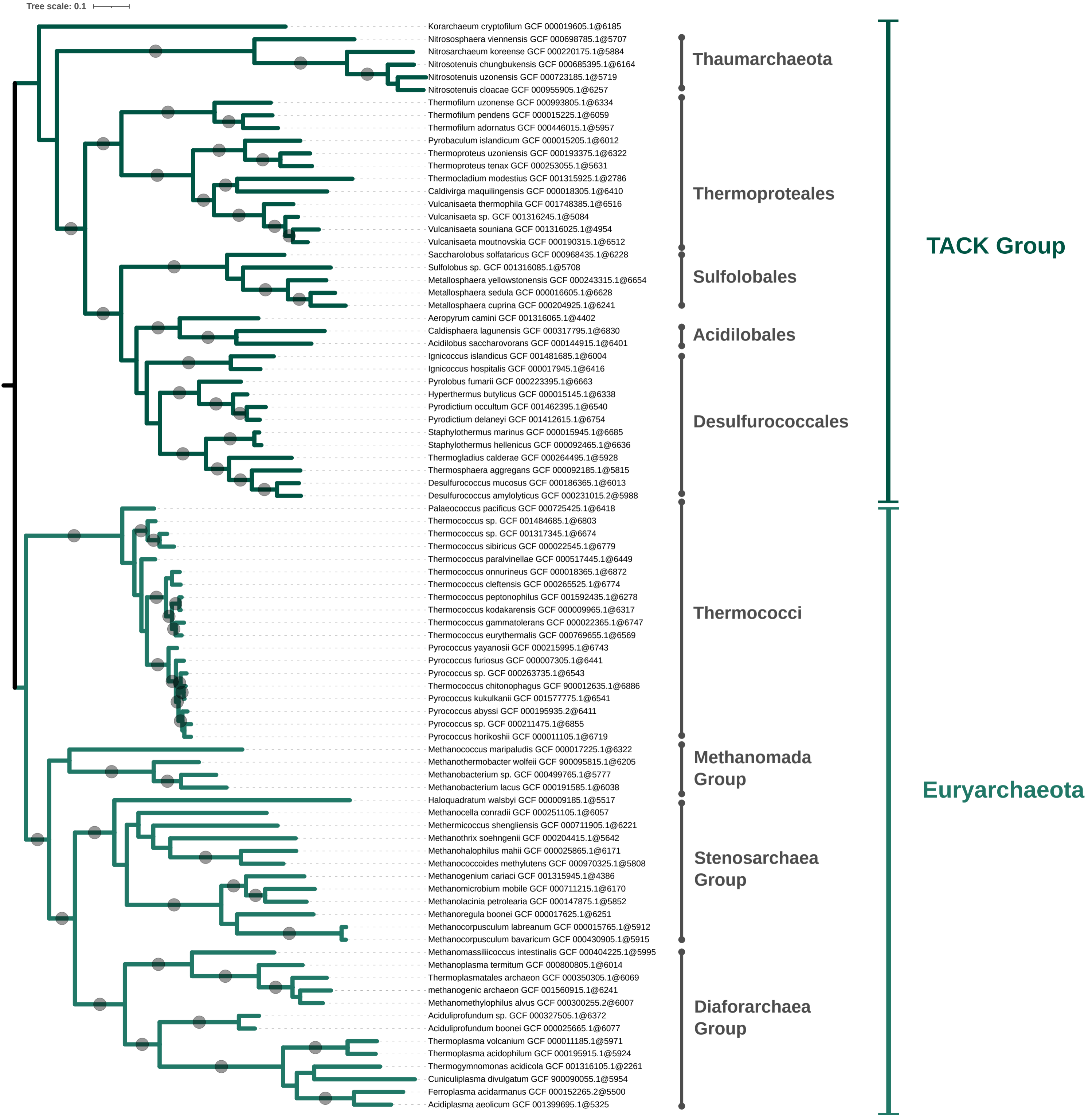

Supplement: Figure S8 — Tree inferred from a supermatrix of concatenated ribosomal proteins (Table 2, I) under the LG4X model using IQ-TREE. Dots on branches indicate maximum bootstrap support values (100%). [file peerj-09-11348-s008.pdf]

**Jl threshold estimation**

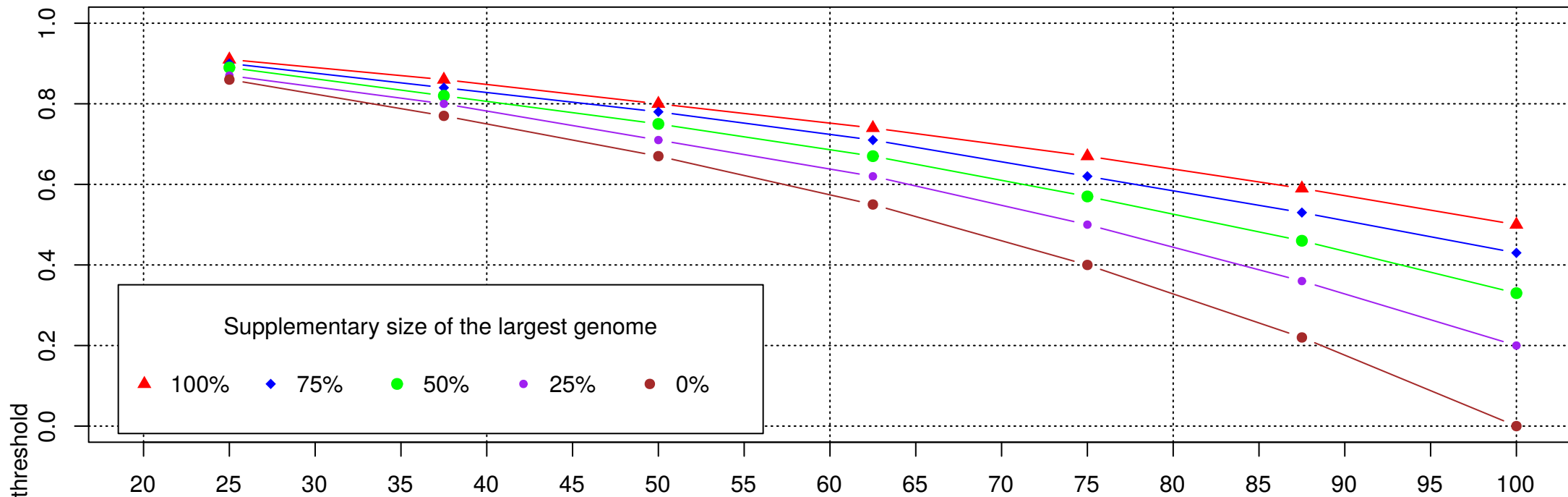

**IGF threshold estimation**

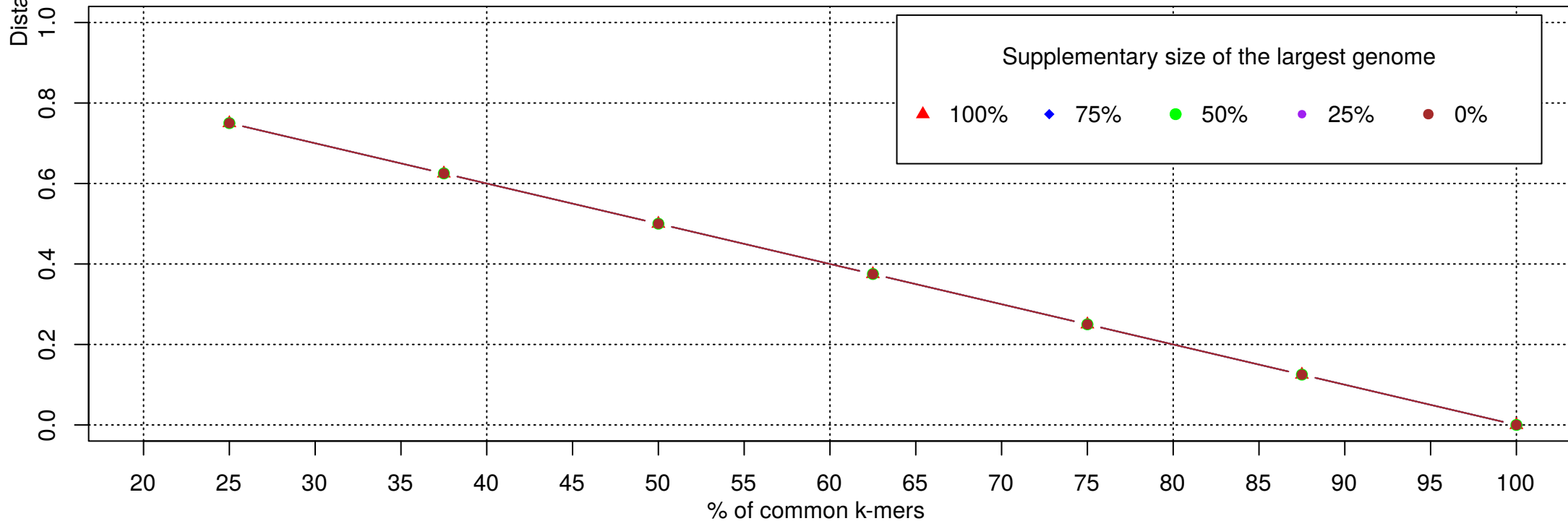

Supplement: Figure S9 — The percentage of common k-mers is given from the smallest genome perspective, i.e., 25% of common k-mers means that 25% of all k-mers from the smallest genome are in common with the largest genome. [file peerj-09-11348-s009.pdf]

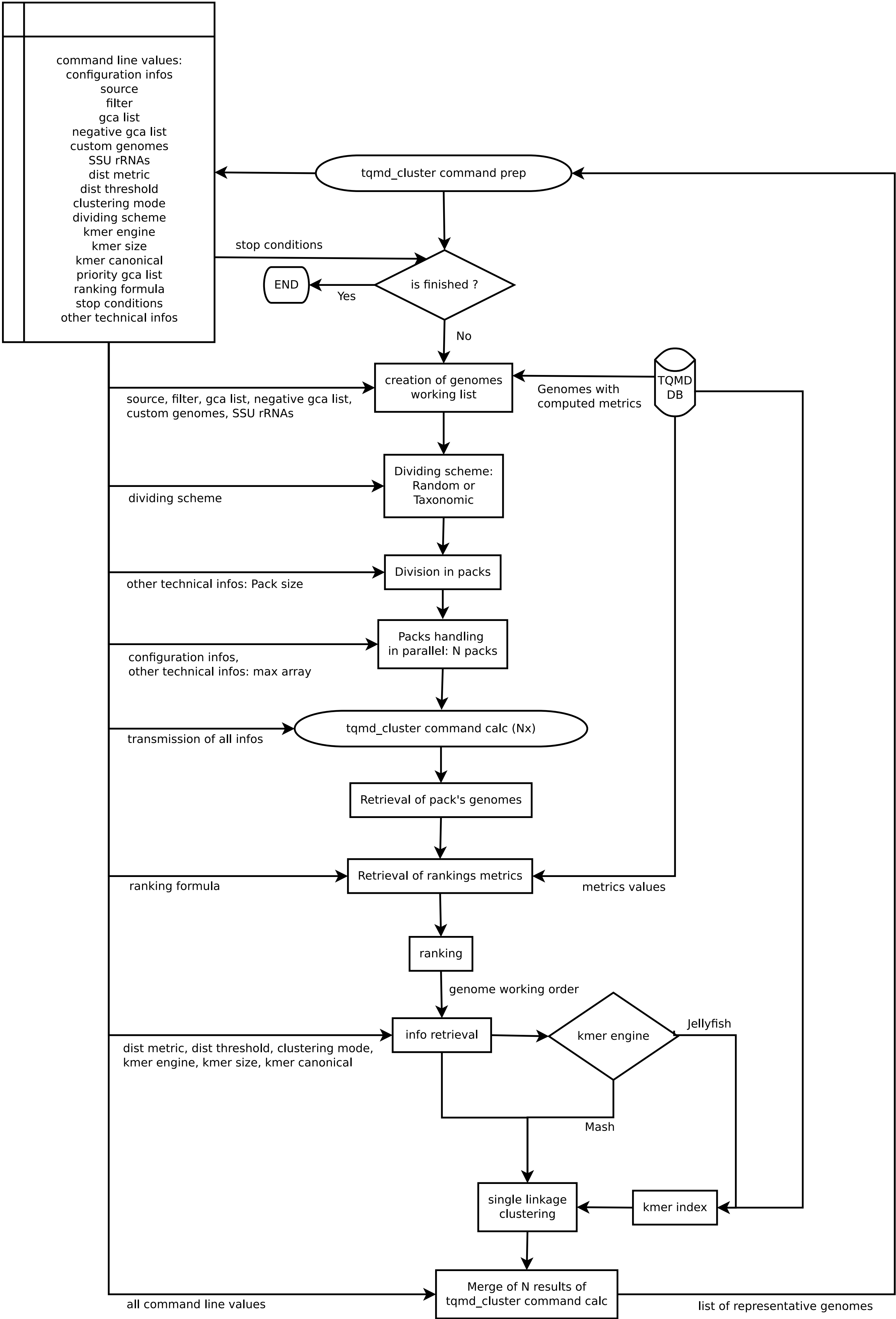

Supplement: Figure S10 [file peerj-09-11348-s010.pdf]
