# Supplementary material for: ToRQuEMaDA: tool for retrieving queried Eubacteria, metadata and dereplicating assemblies
Source: PeerJ. 2021 May 5;9:e11348. doi: 10.7717/peerj.11348 (PMC8106394; doi:10.7717/peerj.11348)
Supplement: Figure S3 — Tree inferred from a supermatrix of concatenated ribosomal proteins (Table 2, D) under the LG4X model using IQ-TREE. Dots on branches indicate maximum bootstrap support values (100%). [file peerj-09-11348-s003.pdf]

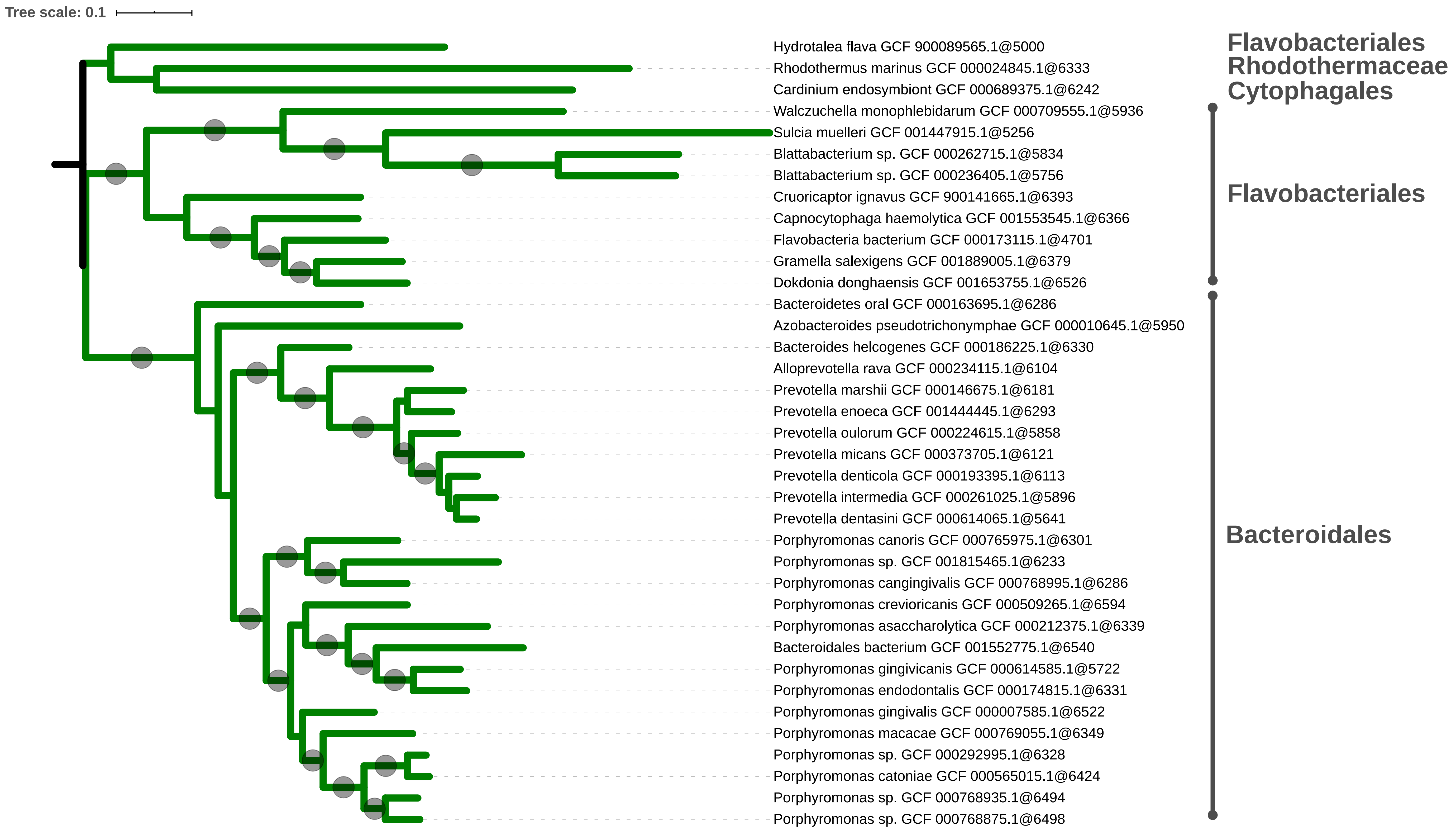
Tree scale: 0.1
Hydrotalea flava GCF 900089565.1@5000
Rhodothermus marinus GCF 000024845.1@6333
Cardinium endosymbiont GCF 000689375.1@6242
Walczuchella monophlebidarum GCF 000709555.1@5936
Sulcia muelleri GCF 001447915.1@5256
Blattabacterium sp. GCF 000262715.1@5834
Blattabacterium sp. GCF 000236405.1@5756
Cruoricaptor ignavus GCF 900141665.1@6393
Capnocytophaga haemolytica GCF 001553545.1@6366
Flavobacteria bacterium GCF 000173115.1@4701
Gramella salexigens GCF 001889005.1@6379
Dokdonia donghaensis GCF 001653755.1@6526
Bacteroidetes oral GCF 000163695.1@6286
Azobacteroides pseudotrichonymphae GCF 000010645.1@5950
Bacteroides helcogenes GCF 000186225.1@6330
Alloprevotella rava GCF 000234115.1@6104
Prevotella marshii GCF 000146675.1@6181
Prevotella enoeca GCF 001444445.1@6293
Prevotella oulorum GCF 000224615.1@5858
Prevotella micans GCF 000373705.1@6121
Prevotella denticola GCF 000193395.1@6113
Prevotella intermedia GCF 000261025.1@5896
Prevotella dentasini GCF 000614065.1@5641
Porphyromonas canoris GCF 000765975.1@6301
Porphyromonas sp. GCF 001815465.1@6233
Porphyromonas cangingivalis GCF 000768995.1@6286
Porphyromonas crevioricanis GCF 000509265.1@6594
Porphyromonas asaccharolytica GCF 000212375.1@6339
Bacteroidales bacterium GCF 001552775.1@6540
Porphyromonas gingivicanis GCF 000614585.1@5722
Porphyromonas endodontalis GCF 000174815.1@6331
Porphyromonas gingivalis GCF 000007585.1@6522
Porphyromonas macacae GCF 000769055.1@6349
Porphyromonas sp. GCF 000292995.1@6328
Porphyromonas catoniae GCF 000565015.1@6424
Porphyromonas sp. GCF 000768935.1@6494
Porphyromonas sp. GCF 000768875.1@6498
Flavobacteriales
Rhodothermaceae
Cytophagales
Flavobacteriales
Bacteroidales
