# Supplementary material for: ToRQuEMaDA: tool for retrieving queried Eubacteria, metadata and dereplicating assemblies
Source: PeerJ. 2021 May 5;9:e11348. doi: 10.7717/peerj.11348 (PMC8106394; doi:10.7717/peerj.11348)
Supplement: Figure S5 — Tree inferred from a supermatrix of concatenated ribosomal proteins (Table 2, F) under the LG4X model using IQ-TREE. Dots on branches indicate maximum bootstrap support values (100%). [file peerj-09-11348-s005.pdf]

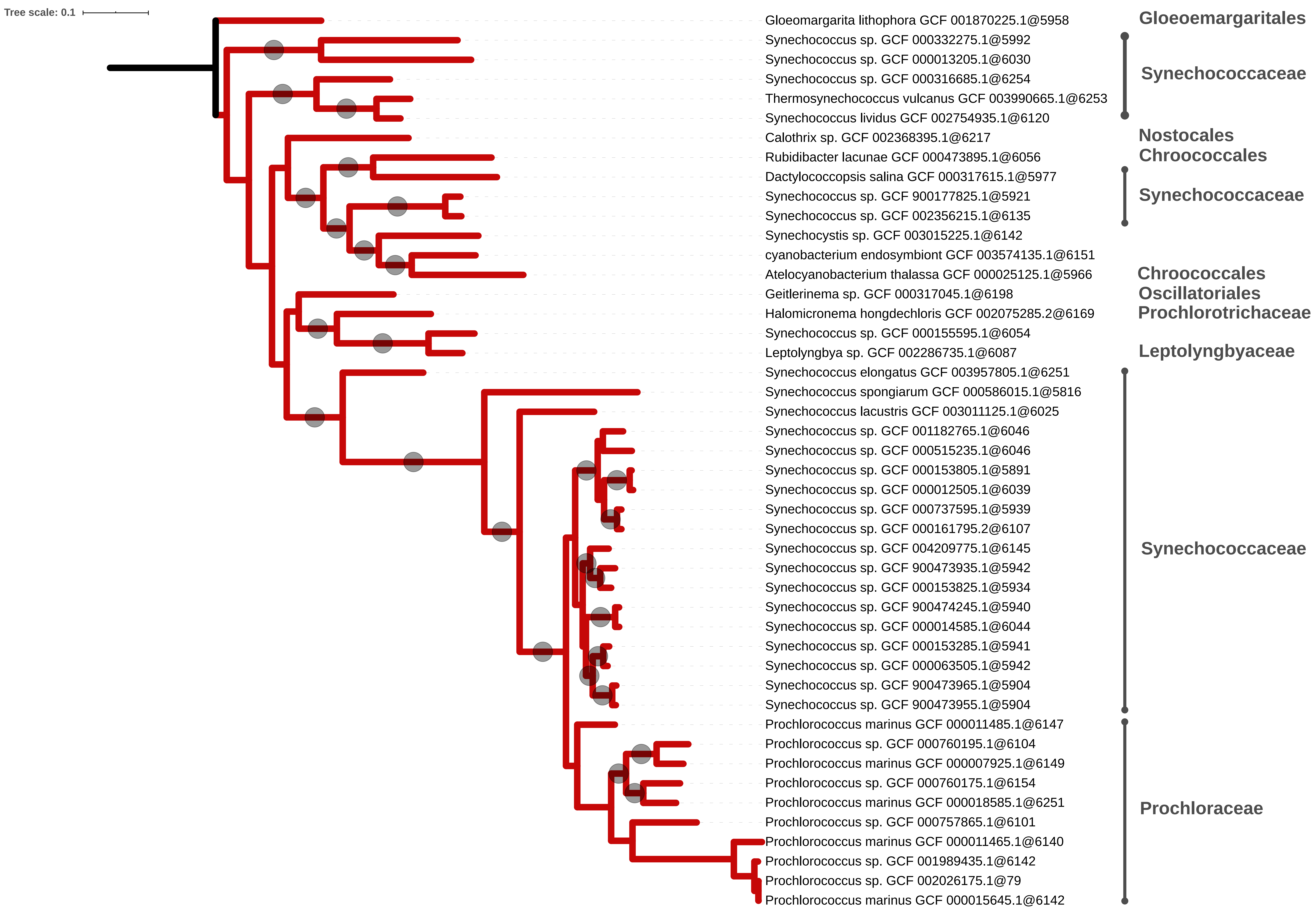
Tree scale: 0.1
Gloeomargarita lithophora GCF 001870225.1@5958
Synechococcus sp. GCF 000332275.1@5992
Synechococcus sp. GCF 000013205.1@6030
Synechococcus sp. GCF 000316685.1@6254
Thermosynechococcus vulcanus GCF 003990665.1@6253
Synechococcus lividus GCF 002754935.1@6120
Calothrix sp. GCF 002368395.1@6217
Rubidibacter lacunae GCF 000473895.1@6056
Dactylococcopsis salina GCF 000317615.1@5977
Synechococcus sp. GCF 900177825.1@5921
Synechococcus sp. GCF 002356215.1@6135
Synechocystis sp. GCF 003015225.1@6142
cyanobacterium endosymbiont GCF 003574135.1@6151
Atelocyanobacterium thalassa GCF 000025125.1@5966
Geitlerinema sp. GCF 000317045.1@6198
Halomicronema hongdechloris GCF 002075285.2@6169
Synechococcus sp. GCF 000155595.1@6054
Leptolyngbya sp. GCF 002286735.1@6087
Synechococcus elongatus GCF 003957805.1@6251
Synechococcus spongiarum GCF 000586015.1@5816
Synechococcus lacustris GCF 003011125.1@6025
Synechococcus sp. GCF 001182765.1@6046
Synechococcus sp. GCF 000515235.1@6046
Synechococcus sp. GCF 000153805.1@5891
Synechococcus sp. GCF 000012505.1@6039
Synechococcus sp. GCF 000737595.1@5939
Synechococcus sp. GCF 000161795.2@6107
Synechococcus sp. GCF 004209775.1@6145
Synechococcus sp. GCF 900473935.1@5942
Synechococcus sp. GCF 000153825.1@5934
Synechococcus sp. GCF 900474245.1@5940
Synechococcus sp. GCF 000014585.1@6044
Synechococcus sp. GCF 000153285.1@5941
Synechococcus sp. GCF 000063505.1@5942
Synechococcus sp. GCF 900473965.1@5904
Synechococcus sp. GCF 900473955.1@5904
Prochlorococcus marinus GCF 000011485.1@6147
Prochlorococcus sp. GCF 000760195.1@6104
Prochlorococcus marinus GCF 000007925.1@6149
Prochlorococcus sp. GCF 000760175.1@6154
Prochlorococcus marinus GCF 000018585.1@6251
Prochlorococcus sp. GCF 000757865.1@6101
Prochlorococcus marinus GCF 000011465.1@6140
Prochlorococcus sp. GCF 001989435.1@6142
Prochlorococcus sp. GCF 002026175.1@79
Prochlorococcus marinus GCF 000015645.1@6142
Gloeoemargaritales
Synechococcaceae
Nostocales
Chroococcales
Synechococcaceae
Chroococcales
Oscillatoriales
Prochlorotrichaceae
Leptolyngbyaceae
Synechococcaceae
Prochloraceae
