# Supplementary material for: ToRQuEMaDA: tool for retrieving queried Eubacteria, metadata and dereplicating assemblies
Source: PeerJ. 2021 May 5;9:e11348. doi: 10.7717/peerj.11348 (PMC8106394; doi:10.7717/peerj.11348)
Supplement: Table S1 — JI-based (direct) analyses were run using a distance threshold of 0.84, whereas IGF-based (direct) analyses used a threshold of 0.66. All analyses were run on 63,863 RefSeq Bacteria using the loose clustering mode. [file peerj-09-11348-s011.docx]

| pack size | # representatives | |
| --- | --- | --- |
|  | JI-d | IGF-d |
| 200 | 836 | 702 |
| 100 | 874 | 866 |
| 50 | 966 | 1197 |
| 25 | 1041 | 1387 |
