# Supplementary material for: ToRQuEMaDA: tool for retrieving queried Eubacteria, metadata and dereplicating assemblies
Source: PeerJ. 2021 May 5;9:e11348. doi: 10.7717/peerj.11348 (PMC8106394; doi:10.7717/peerj.11348)
Supplement: Supplemental Information 1 [file peerj-09-11348-s012.docx]

## ToRQuEMaDA: Tool for Retrieving Queried Eubacteria, Metadata and Dereplicating Assemblies - Supplementary material

Raphaël R. Léonard^a,b^, Marie Leleu^b,c^, Mick Van Vlierberghe^b^, Luc Cornet^b,d^, Frédéric Kerff^a^, Denis Baurain*^b^

^a^ InBioS – Centre d’Ingénierie des Protéines, Université de Liège, Belgium

^b^ InBioS – PhytoSYSTEMS, Eukaryotic Phylogenomics, Université de Liège, Belgium

^c^ Univ. Lille, CNRS, UMR 8576 – UGSF – Unité de Glycobiologie Structurale et Fonctionnelle, F-59000 Lille, France

^d^ Mycology and Aerobiology - Sciensano, Service Public Fédéral - Bruxelles, Belgium

* Corresponding author

### Comparison with Assembly-Dereplicator

A-D is a program that is more recent than dRep but, as of August 2020, not yet published; its last update dates from November 2019. Its main advantage is ease of use, since it is a simple (no-installation) script that only needs Mash as a prerequisite. A-D takes as input the path to a folder containing the genomes to be dereplicated and rearranges them randomly and separated into smaller packs (500 genomes per pack by default). The next step is the clustering of each pack serially using Mash. A-D stops as soon as it cannot dereplicate at least one genome from the current pack. Since it was compatible with our grid computer, we tried to test A-D (v0.1.0) with all prokaryotic RefSeq genomes, so as to mimic how TQMD is supposed to work in addition to the two datasets used with dRep.

For each of the two smaller datasets, A-D required only one CPU and took ten minutes when not partially crashing. Each dataset had to be relaunched several times due to A-D not finding the path to Mash for each pack. In the following, the TQMD results are the same as those reported for the dRep comparison. For Bacteroidetes, A-D selected 798 representatives (519 species), of which 704 were in common with TQMD, which represents 498 species in common. For the Streptomycetales, A-D selected 435 representatives (190 species), of which 408 were in common with TQMD, which represents 180 species (details given in Table S2).

In March 2019, all RefSeq prokaryotic genomes amounted to 112,254 genomes. We launched several A-D runs which all stopped after one hour and failed to dereplicate more than 5,000 genomes despite an increasingly lenient threshold (details given in Table S3). Investigation of the results revealed that this time the problem was not due to A-D not finding Mash but caused by the heuristics implemented in A-D.

| dataset | starting | | TQMD - JELLYFISH k12 | | | Assembly-Dereplicator | | | intersection | |
| --- | --- | --- | --- | --- | --- | --- | --- | --- | --- | --- |
|  | # gen. | # spec. | # repr. | # spec. | h.CPU | # repr. | # spec. | h.CPU | # repr. | # spec. |
| Bacteroidetes | 1127 | 528 | 789 | 517 | 60 | 798 | 519 | 0.1 | 704 | 498 |
| Streptomycetales | 648 | 220 | 486 | 207 | 40 | 435 | 190 | 0.1 | 408 | 180 |

**Supplementary Table 2. Performance comparison between TQMD and Assembly-Dereplicator on two smaller datasets.** The column titles and the TQMD results are taken from Table 7.

| dist. threshold | # representatives | # derepl. packs |
| --- | --- | --- |
| 0.01 | 111,855 | 4 |
| 0.10 | 110,160 | 9 |
| 0.20 | 111,596 | 3 |
| 0.30 | 112,254 | 1 |
| 0.40 | 108,774 | 8 |
| 0.50 | 111,755 | 2 |

**Supplementary Table 3. Attempts at dereplicating all RefSeq Bacteria (releases 79+92, 112,254 genomes) using Assembly-Dereplicator.** Analyses were run using 6 different distance thresholds and the default pack size of 500 (225 packs).

Apparently, A-D does not work with very large and non-homogeneous groups of genomes. We did not investigate further the script with large non-homogenous datasets since our tests clearly showed that it cannot be compared, in its present state, with TQMD. A-D can dereplicate smaller sets of homogeneous genomes (such as the Cyanobacteria or the aforementioned Bacteroidetes and Streptomycetales) provided the bug with Mash not being recognized is solved. Yet, drawing on our own tests with TQMD (see main text), our intuition is that the A-D approach based on a random splitting of the genomes to dereplicate, if appropriate when working with homogeneous genomes, are likely to be inefficient when it comes to non-homogeneous datasets. Moreover, the stop conditions of the iterative heuristics obviously lead A-D to get stuck very easily.
